# Second-line chemotherapy for the treatment of metastatic pancreatic cancer after first-line gemcitabine-based chemotherapy: a network meta-analysis

**DOI:** 10.18632/oncotarget.25639

**Published:** 2018-07-03

**Authors:** Chiara Citterio, Michela Baccini, Elena Orlandi, Camilla Di Nunzio, Luigi Cavanna

**Affiliations:** ^1^ Onco-Haematology Department, Hospital Guglielmo da Saliceto, 29121 Piacenza, Italy; ^2^ Department of Statistics, Informatics, Applications “G. Parenti”, Università di Firenze, 50134 Florence, Italy

**Keywords:** pancreatic cancer, second-line chemotherapy, oxaliplatin, irinotecan, gemcitabine based chemotherapy

## Abstract

Guidelines for treatment of metastatic pancreatic cancer recommend a second line based on Fluoropyrimidine (FP) alone or in combination with Oxaliplatin (OXA) or Irinotecan (IRI) after a first line treatment based on Gemcitabine (GEM). We conducted a Bayesian network meta-analysis to compare currently available therapies to treat metastatic pancreatic cancer in the second line, considering as efficacy measures overall survival (OS) and progression free survival (PFS). Published randomized trials were identified using electronic databases (MEDLINE, PubMed, https://clinicaltrials.gov/ and American Society of clinical oncology). 8 studies met the inclusion criteria for a total of 1,587 patients and 7 different therapeutic schemes. The results suggested that the use of IRI-FP-Folinic Acid scheme in the second-line treatment of metastatic pancreatic cancer may offer a benefit in terms of OS and PFS for patients not previously treated with these drugs.

## INTRODUCTION

Pancreatic cancer still has a poor prognosis, and progress in the treatment of this disease is poor. Several studies have been conducted regarding the first-line therapy [1], however the second-line remains a field to be explored. Standard first-line therapies are combinations of Gemcitabine (GEM) based chemotherapy, such as GEM plus Nab-paclitaxel or, for the best performing patients, the FOLFIRINOX regimen containing 5-fluorouracil (5-FU), Oxaliplatin (OXA), Irinotecan (IRI) and folinic acid (FA). The choice between these two regimens poses a challenging problem: for patients candidate to receive a second-line chemotherapy, the selection depends in part on their previous treatment. Recently, new drugs, such as nanoliposomial IRI in combination with 5-FU and folinic acid (FA), have been approved by the Food and Drug Administration (FDA), especially after disease progression on first-line gemcitabine-based therapy. After a progression from the first line containing GEM, the ASCO (American Society of Clinical Oncology) guidelines recommend a second line based on Fluoropyrimidine (FP) alone or in combination with OXA or IRI [2]. Currently there are no randomized trials conducted on a large number of patients indicating which of the proposed therapies are the best from the point of view of efficacy. The aim of this project was to compare, through a Bayesian network meta-analysis of published randomized clinical trials, the currently available therapies to treat metastatic pancreatic cancer in the second line, after a first line treatment based on GEM/GEM combination. We focused on both Overall Survival (OS), and Progression Free Survival (PFS).

## RESULTS

**Figure 1:**
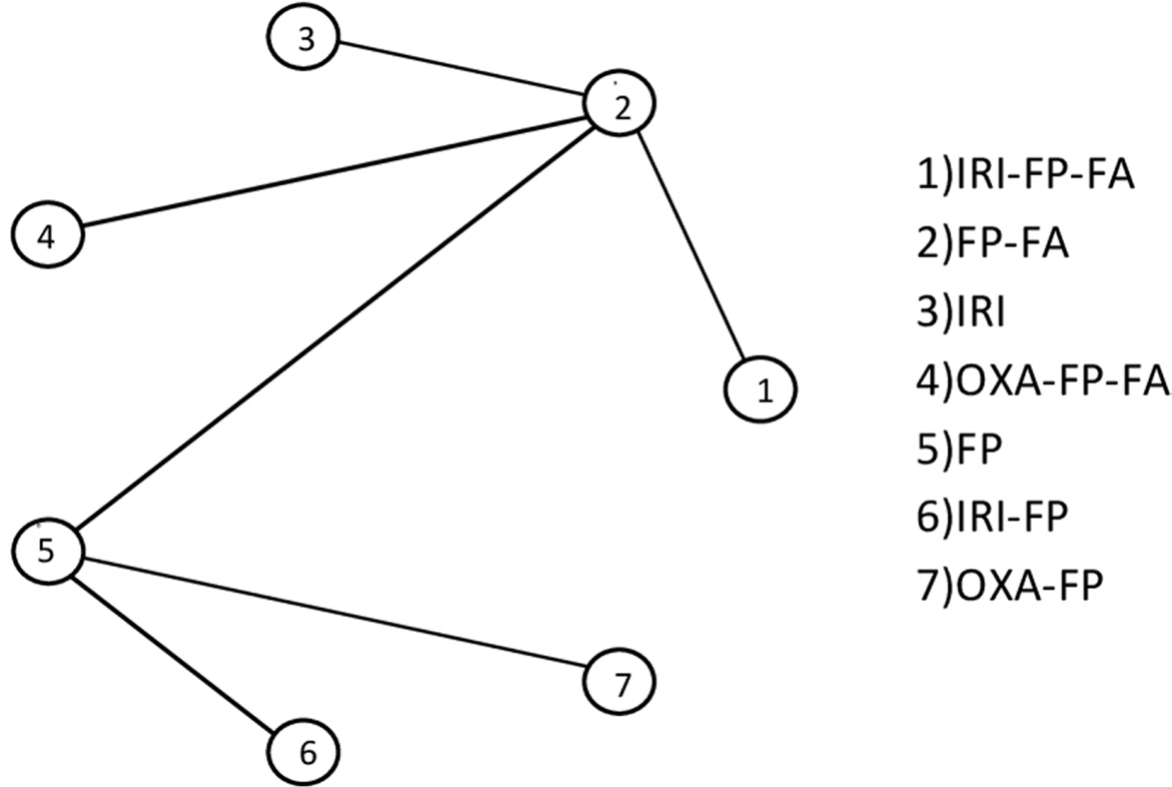
Study network

The 8 studies included in the network involved a total of 1,587 patients (average age 63.7 years, 56.8% males) at the second line therapy after a first line of therapy with gemcitabine or gemcitabine combinations. Only for 3 studies (52.74% of the 1,587 patients) information was available about the exact first line therapy used: 498 patients were treated with gemcitabine monotherapy and 339 with gemcitabine combinations. The patients performance status was reported in 6 studies (84% of the 1,587 patients): patients have a performance status between 0 (502 patients), 1 (741 patients) and 2 (55 patients) (Supplementary Table 1). The second line chemotherapy drugs studied in various combinations were IRI, FP, folinic acid (FA) and OXA. A total of 7 treatments were compared: IRI + FP + FA (1), FP + FA (2), IRI (3), OXA + FP + FA (4), FP (5), IRI + FP (6), FP + OXA (7) (Figure 1).

We had direct information on six comparisons (Figure 1): the comparison between therapeutic scheme 1 (IRI + FP + FA) and 2 (FP + FA), between 2 and 3 (IRI), between 2 and 4 (OXA + FP + FA), 2 and 5 (FP), 5 and 6 (IRI + FP) and 5 and 7 (OXA + FP).

For comparisons 2–4, 5–2 and 5–6, data from two studies were available (an individual fixed effects meta-analyzes was performed; since the comparison involved only two studies, it was not reported), while data from only one study were available for the others comparisons.

### Results from the network meta-analysis

**Figure 2:**
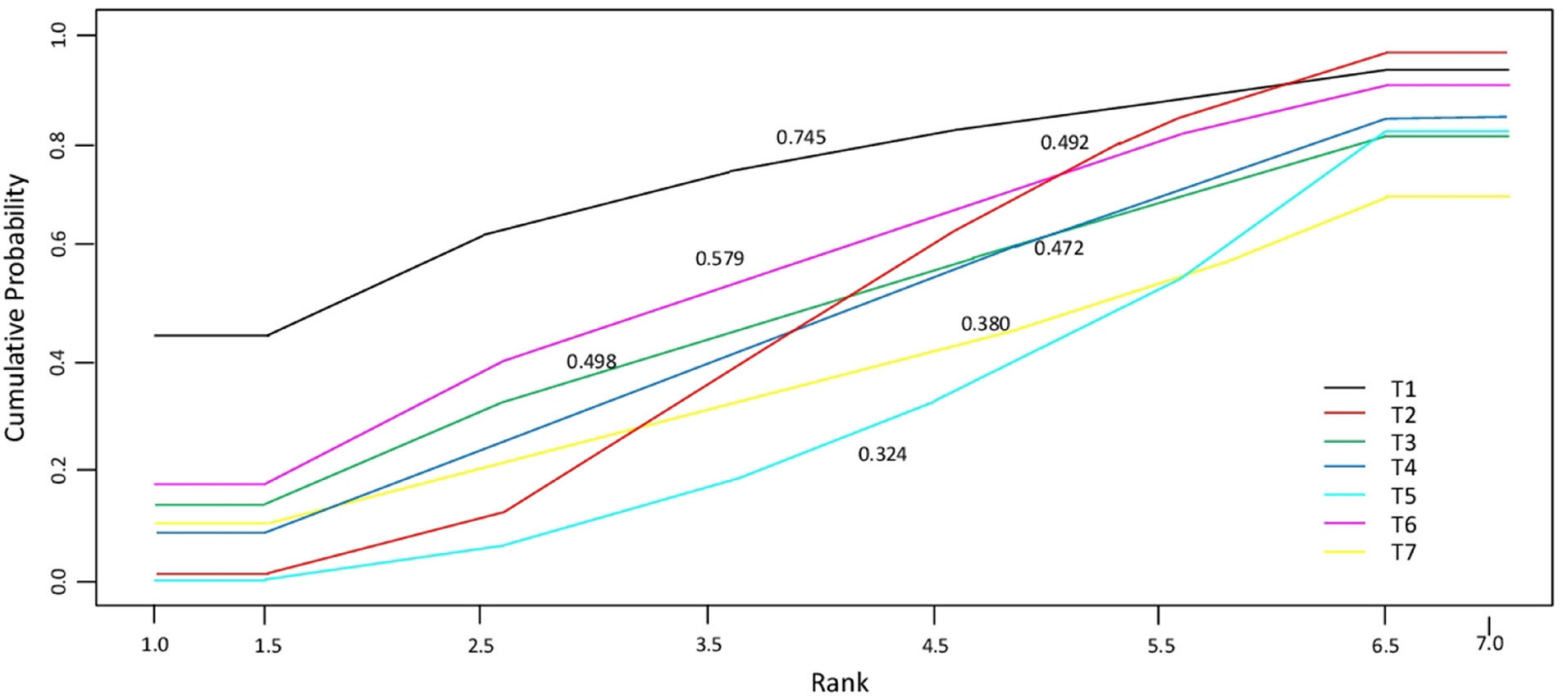
Cumulative probability of the treatment rank and SUCRA for the 7 treatments from the network meta-analysis on OS

**Figure 3:**
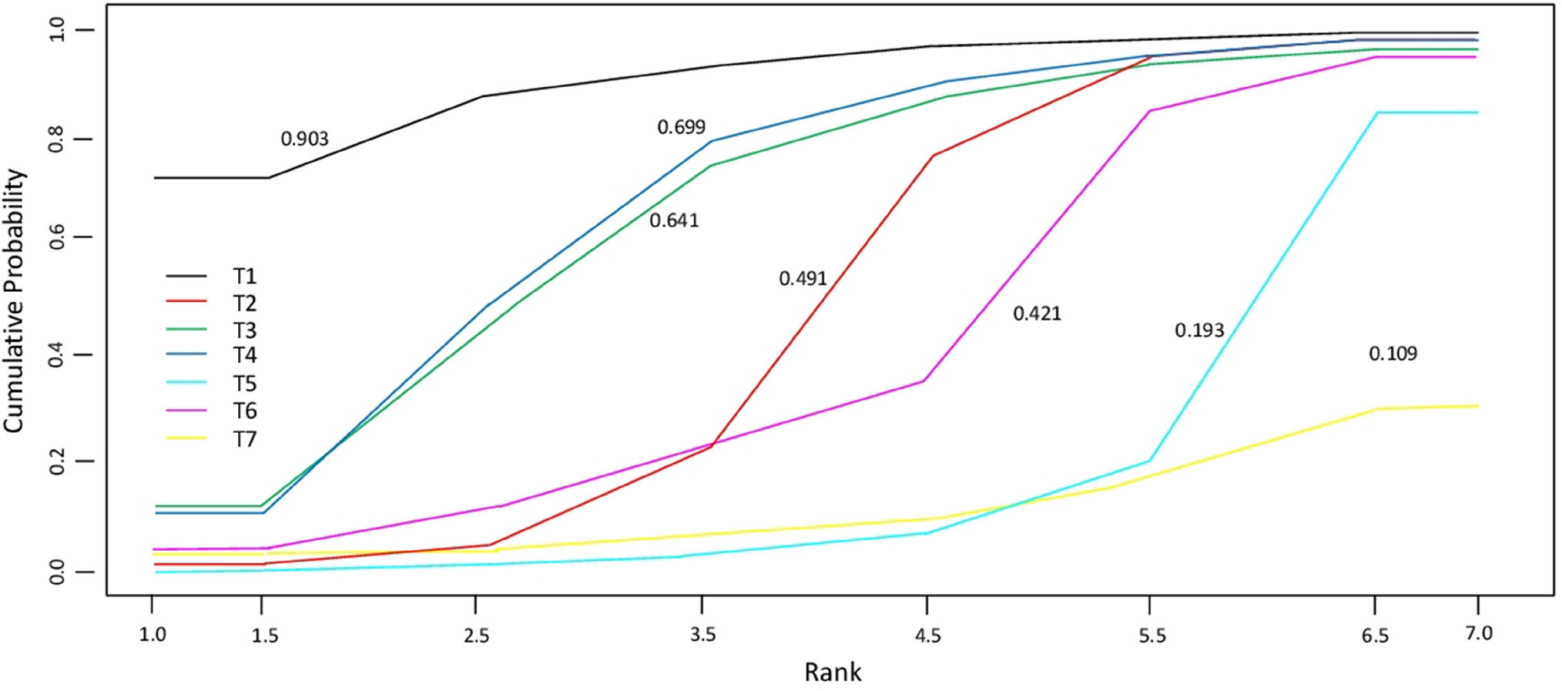
Cumulative probability of the treatment rank and SUCRA for the 7 treatments from the network meta-analysis on PFS

In Table 1 the estimates of the pairwise comparisons arising from the network meta-analyses are reported, separately for OS and PFS. The combination IRI-FP-FA had better performance than all the other treatments, especially in respect of FP and OXA-FP, both in terms of OS and PFS. Differences among treatments were more evident when focusing on PFS. Results for IRI-FP changed according to the outcome: when comparing IRI-FP with OXA-FP-FA, IRI-FP was better in terms of OS while OXA-FP-FA was better in terms of PFS; when comparing IRI-FP with IRI, IRI-FP was better in term of OS while IRI was better in terms of PFS. The median of the I^2^ distribution was around 85% for OS (90% Crl: 34,2%–97,9%) and close to 55% for PFS (90% Crl: 0.99%–97.7%). The combination IRI-FP-FA resulted as having the largest probability of being the best and the lower posterior average rank, in particular when the focus was on PFS (Table 2). FP was the worst treatment in terms of best and average rank in both analyses. These results are confirmed by the analysis of the cumulative probabilities of the treatment rank (Figure 2). When considering OS the most effective therapeutic combinations (SUCRA = 75%) were treatment schemes containing Irinotecan: IRI-FP-FA, followed by IRI-FP (58%), and IRI The SUCRA for the remaining treatments was similar, with values varying between 47% and 50%. As far as PFS is concerned (Figure 3), the best treatment was IRI-FP-FA (90%), followed by OXA-FP-FA plus IRI, both having similar performance in terms of SUCRA (70% and, 69%, respectively). Also in this setting, the worst results were observed for the combinations OXA-FP and FP (11% and 20% respectively).

**Table 1:** Estimated HR (posterior mean, 90% Credibility Interval) for all pairwise comparisons from the network meta-analysis on OS and PFS

Regimen	Mean	90% CI
**OS**		
IRI-FP-FA/FP-FA	0.67	0.22–2.14
IRI-FP-FA/IRI	0.68	0.14–3.49
IRI-FP-FA/OXA-FP-FA	0.65	0.15–2.61
IRI-FP-FA/FP	0.55	0.13–2.34
IRI-FP-FA/IRI-FP	0.74	0.14–3.78
IRI-FP-FA/OXA-FP	0.57	0.09–3.53
FP-FA/IRI	1.01	0.33–3.19
FP-FA/OXA-FP-FA	0.96	0.41–2.12
FP-FA/FP	0.83	0.35–1.93
FP-FA/IRI-FP	1.09	0.34–3.56
FP-FA/OXA-FP	0.84	0.20–3.49
IRI/OXA-FP-FA	0.95	0.22–3.78
IRI/FP	0.81	0.19–3.39
IRI/IRI-FP	1.08	0.21–5.64
IRI/OXA-FP	0.84	0.13–5.16
OXA-FP-FA/FP	0.85	0.20–2.89
OXA-FP-FA/IRI-FP	1.14	0.28–5.00
OXA-FP-FA/OXA-FP	0.88	0.18–4.66
FP/IRI-FP	1.34	0.59–3.03
FP/OXA-FP	1.03	0.33–3.22
IRI-FP/OXA-FP	0.77	0.19–3.13
**PFS**		
IRI-FP-FA/FP-FA	0.56	0.29–1.06
IRI-FP-FA/IRI	0.69	0.28–1.70
IRI-FP-FA/OXA-FP-FA	0.70	0.31–1.52
IRI-FP-FA/FP	0.37	0.17–0.87
IRI-FP-FA/IRI-FP	0.48	0.19–1.27
IRI-FP-FA/OXA-FP	0.31	0.11–0.90
FP-FA/IRI	1.23	0.66–2.32
FP-FA/OXA-FP-FA	1.25	0.76–1.97
FP-FA/FP	0.66	0.40–1.14
FP-FA/IRI-FP	0.86	0.44–1.77
FP-FA/OXA-FP	0.55	0.25–1.30
IRI/OXA-FP-FA	1.01	0.45–2.18
IRI/FP	0.53	0.24–1.23
IRI/IRI-FP	0.70	0.28–1.82
IRI/OXA-FP	0.45	0.17–1.28
OXA-FP-FA.FP	0.53	0.27–1.11
OXA-FP-FA/IRI-FP	0.69	0.31–1.67
OXA-FP-FA/OXA-FP	0.44	0.18–1.20
FP/IRI-FP	1.30	0.81–2.10
FP/OXA-FP	0.84	0.44–1.58
IRI-FP/OXA-FP	0.65	0.30–1.43

**Table 2:** Posterior probability of being the best treatment and average rank for the 7 compared treatments, from the network meta-analysis on OS and PFS

Regimen	Mean	Median	Best.perc	90% CI
**OS**				
IRI-FP-FA	2.53	2	1–7	45.64
FP-FA	4.05	4	2–6	1.72
IRI	4.01	4	1–7	14.43
OXA-FP-FA	4.14	4	1–7	8.73
FP	5.06	5	2–7	1.02
IRI-FP	3.53	3	1–7	17.87
OXA-FP	4.68	5	1–7	10.58
**PFS**				
IRI-FP-FA	1.56	1	1-4	72.24
FP-FA	4.05	4	3–6	0.3
IRI	2.92	3	1–6	12.25
OXA-FP-FA	2.81	3	1–6	9.86
FP	5.84	6	4–7	0.16
IRI-FP	4.47	5	2–7	3.69
OXA-FP	6.35	7	3–7	1.49

## DISCUSSION

The aim of this study was to combine in a network-meta-analysis published results comparing OS and PFS in patients with metastatic pancreatic cancer treated with different second line therapeutic schemes. The analysis included also schemes that did not contain OXA or IRI, commonly used in clinical practice, especially in Asia [12].

The results suggest that the use of IRI-FP-FA may offer a benefit in terms of both OS and PFS for patients not previously treated with these drugs.

A recent systematic review [13], collected 24 among observational, retrospective, prospective, randomized, comparative and non-comparative studies, comparing FP-based chemotherapy with either OXA or IRI. The conclusion of this study highlight the lack of significant differences in terms of OS and PFS when using a second line therapy based on IRI or OXA. Our meta-analysis. which included only randomized trials and focused on a larger set of therapies (FP-based chemotherapy with either OXA, IRI and FP-based therapies alone), found a difference.

Sonbol *et al.* [14] performed a meta-analysis on second-line treatments in patients with pancreatic ductal adenocarcinoma, with the aim of investigating the effectiveness of adding OXA or various IRI formulations to FP after first-line treatment progression. They found that the combination of FP with IRI formulations was the appropriate next line of treatment upon progression after gemcitabine-based chemotherapy regimens, confirming the overall results of our current meta-analysis.

Despite the recent advances in the general overview of cancer therapy, the prognosis of advanced pancreatic cancer still remains poor. For more than 15 years, gemcitabine monotherapy has been the cornerstone of first-line treatment; a new formulation of paclitaxel (nab-paclitaxel) was recently used in combination with gemcitabine as a first-line regimen, with a greater PFS (5.5 versus 3.7 months) [17]. Many recent analyses have examined the use of second-line therapies after this regime: in the global phase III trial MPACT patient receiving second-line therapy after nab-Paclitaxel/GEM experienced a longer median OS than those who did not (12.8 versus 6.3 months, respectively) [18]. The longest total OS values were observed in patients who received first-line nab-Paclitaxel/GEM followed by fluoropyrimidine-containing second-line regimens (median, 13.5 months); the small number who received second-line FOLFIRINOX reached a median total OS of 15.7 months [18]. Another retrospective analysis led to similar findings, with a median OS of 13.5 months in patients who received second-line treatment after first-line nab-P/Gem (with a benefit with the FOLFIRINOX regimen, 13,8 months of total OS, versus 13,2 with FOLFIRI and 12,8 with FOLFOX/XELOX regimen) [19, 20].

It must be emphasized that most of the studies included in our network have been conducted in China and Japan. This is an important point to bear in mind when interpreting the results, since in the literature there is evidence of heterogeneity between Western and Eastern countries in cancer treatment and in patient’s response [15, 16].

Our analysis indicates the presence of a certain heterogeneity between studies, in particular for the OS results, and mainly due to the comparison FP-FA and OXA-FP-FA. However, given the small number of studies available for each comparison, it is difficult to make hypothesis about the origin of this heterogeneity. It must be underlined that in this review patients’ quality of life and toxicities of the treatment schemes were not considered and that are available only limitated informations about best treatment sequence because only for 3 studies the information about first line therapy are available.

The main limitation of our analysis is the small number of studies that we included in our network, which reflects the fact that in the literature there are only few papers reporting results from clinical trials comparing second-line therapies for pancreatic cancer. Therefore, in order to obtain a complete answer to our research question, further randomized studies are needed.

A better understanding of how an effective first line treatment may influence clinical benefit in subsequent treatments is essential. Furthermore, additional indication for second line treatment will depend on enhanced identification of biologic predictors of second line therapy benefit, development of more active regimens, and investigation of the specific toxicity of each regimen.

However, our analysis recommends FP, FA and IRI formulation for the second line treatment after first line GEM/GEM combination, based on OS and PFS evaluation; since there are no data regarding the best second line after FOLFIRINOX first line therapy, further exploration is warranted.

## MATERIALS AND METHODS

### Study identification

**Figure 4:**
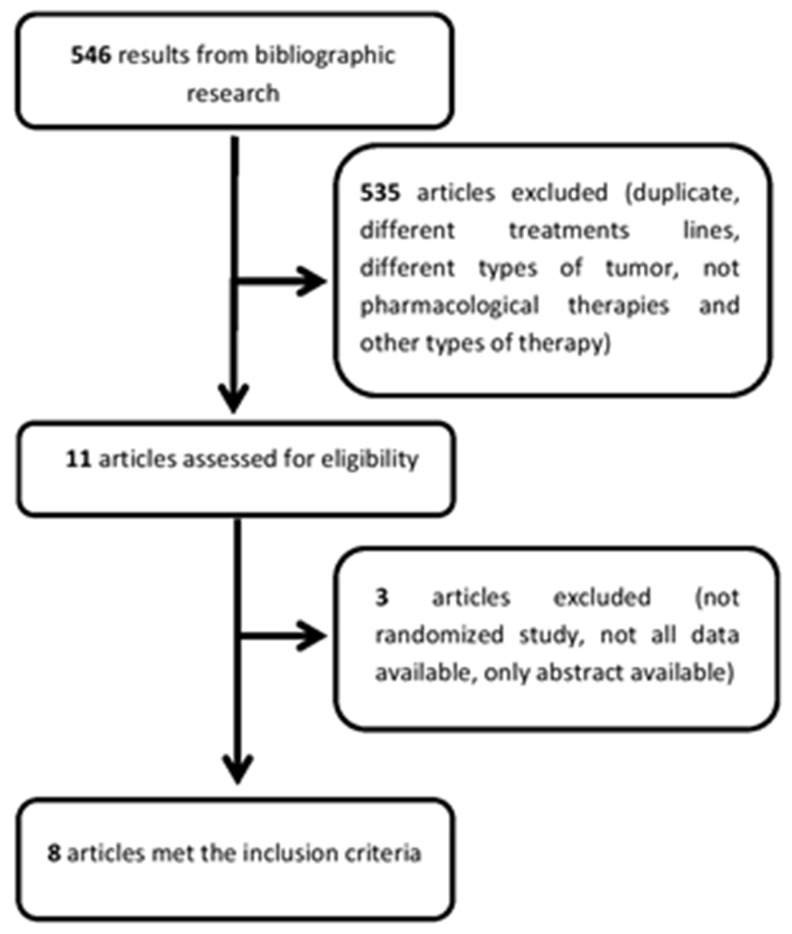
Flow chart illustrating the result of the online search and articles selection

Articles, published between 2009 and 2017, were sought in: MEDLINE, PubMed, https://clinicaltrials.gov/ and American Society of clinical oncology (ASCO). The search was based on the following keywords: “pancreatic cancer”, “second line” and “chemotherapy”. Initially, the online search led to 399 results from PubMed-MEDLINE, 34 from https://clinicaltrials.gov/ and 113 from ASCO. After removing duplicates, studies on different treatment lines, different types of cancer, first line not GEM/GEM combination based, not pharmacological therapies and other experimental therapy, a total of 11 studies were considered eligible for further review (Figure 4). Two individual reviewers (E.O. and L.C.) read the articles and extracted from them relevant information, including patient characteristics, study design, sample size, outcome measures and other study characteristics. Of the 11 selected studies 3 were excluded: one was an observational study [3], one did not provide the values of the Hazard Ratio (HR) [4], for a third study, some data were unknown since only the abstract was available [5]. The remaining 8 studies were randomized, phase 2-3 studies; all reported the number of patients and only 5 their average age. Four studies were conducted in Japan, 1 in China, 1 in Canada, 1 in Germany and 1 involved multiple countries (Supplementary Table 1).

### Statistical analysis

Separate analyses were performed for OS and PFS. We focused on the Hazard Ratio (HR). First, for descriptive purposes, single meta-analyses were performed for each comparison with at least two studies available (not reported). Then, a Bayesian meta-analysis network was conducted. The network meta-analysis allows for simultaneous comparisons between multiple treatments, ranking them according to their effectiveness. Moreover, it permits inference also on comparisons never directly observed in ad hoc clinical trials [6–8]. We adopted a Bayesian approach as it provides more reliable estimates of the variability occurring in the network (heterogeneity and inconsistency) and allows to obtain in a more natural way a ranking of the compared treatments. The analyzed outcomes were Overall Survival (OS), and Progression Free Survival (PFS) (Table 3). In the present study, a model was specified that assumed consistency between direct and indirect evidence, because in the network there were no observed comparisons that could be combined to make indirect inference on a third observed comparison (no closed loops in Figure 1) [6–9] The model accounted for possible heterogeneity among the results conducted on the same comparison. Heterogeneity was measured using the I^2^ statistic [10]. From the network meta-analysis, we obtained a posterior estimate of the HR for each pairwise comparison, with the corresponding 90% Credibility Interval. For each treatment the cumulative probability of the treatment rank was drawn and the area under the defined curve (SUCRA) calculated. The SUCRA, which can also be obtained through a transformation of the average rank, provides a measure of the relative performance of the treatment compared to the others. The greater the value of the SUCRA, i.e. the greater the portion of area under the curve, the better the treatment performance [11]. All statistical analyses were performed using R software (Core Team (2017). R: A language and environment for statistical computing. R Foundation for Statistical Computing, Vienna, Austria. URL https://www.R-project.org/), and the R2WinBUGS package. Codes have been written ad hoc for this analysis.

**Table 3:** OS, PFS and HR for the 8 studies included in the network meta-analysis

Author	Treatment	OS [95% CI]	HR	CI (95%)	se	*p*	PFS [95% CI]	HR	CI (95%)	se	*p*
Gillam *et al.* [21] (2016)	IRI-FP-FA FP-FA (ref.)	6.1 [4.8–8.9] 4.2 [3.3–5.3]	0.67	0.49–0.92	0.16	0.01	3.1 [2.7–4.2] 1.5 [1.4–1.8]	0.56	0.41–0.75	0.15	0.00
Gillam *et al.* [21] (2016)	IRI FP-FA (ref.)	4.9 [4.2–5.6] 4.2 [3.6–4.9]	0.99	0.77–1.28	0.13	0.94	2.7 [2.1–2.9] 1.6 [1.4–1.8]	0.81	0.63–1.04	0.13	0.10
Oettle *et al.* [22] (2014)	FP-FA (ref.) OXA-FP-FA	3.3 [2.7–4.0] 5.9 [4.1–7.4]	0.66	0.48–0.91	0.16	0.01	2.0 [1.6–2.3] 2.9 [2.4–3.2]	0.68	0.50–0.94	0.17	0.02
Gill *et al.* [23] (2016)	FP-FA (ref.) OXA-FP-FA	9.9 [6.7–16.9] 6.1 [3.2–7.1]	1.78	1.08–2.93	0.25	0.02	2.9 [1.7–5.1] 3.1 [1.9–7.2]	1.00	0.66–1.53	0.22	0.99
Ge *et al.* [24] (2014)	FP (ref.) FP-FA	5.5 6.3	0.83	0.66–1.67	0.36	0.08	1.9 3.0	0.86	0.66–1.63	0.33	0.86
Ueno *et al.* [25] (2016)	FP (ref.) FP-FA	6.1 6.3	0.82	0.54–1.22	0.20	0.46	2.7 3.8	0.56	0.37–0.85	0.21	0.00
Ioka *et al.* [26] (2017)	FP (ref.) FP-IRI	5.8 6.8	0.75	0.51–1.09	0.19	0.13	1.9 3.5	0.77	0.53–1.11	0.19	0.02
Mizuno *et al.* [27] (2013)	FP (ref.) FP-IRI	5.9 6.9	0.75	0.51–1.09	0.19	0.13	1.93 3.57	0.77	0.53–1.11	0.19	0.18
Ohkawa *et al.* [28] (2015)	FP OXA-FP (ref.)	6.9 7.4	1.03	0.79–1.34	0.13	0.82	2.8 3.0	0.84	0.65–1.08	0.13	0.18

Abbreviations: OS = Overall Survival, HR = Hazard Ratio, CI = Confidence Interval, se = standard error, *p* = *p*-value, PFS = Progression Free Survival, Ref. = reference treatment.

## SUPPLEMENTARY MATERIALS TABLE



## Abbreviations

(5-FU): 5-fluorouracil
(ASCO): American Society of Clinical Oncology
(FP): Fluoropyrimidine
(FA): folinic acid
(FDA): Food and Drug Administration
(GEM): Gemcitabine
(cGEM): gemcitabine combination
(IRI): Irinotecan
(nd): no data
(OS): Overall Survival
(OXA): Oxaliplatin
(PS): Performance Status
(PFS): Progression Free Survival
