# Supplementary material for: Second-line chemotherapy for the treatment of metastatic pancreatic cancer after first-line gemcitabine-based chemotherapy: a network meta-analysis
Source: Oncotarget. 2018 Jul 3;9(51):29801–9. doi: 10.18632/oncotarget.25639 (PMC6049864; doi:10.18632/oncotarget.25639)
Supplement: Supplementary file 1 [file oncotarget-09-29801-s001.pdf]

Second-line chemotherapy for the treatment of metastatic pancreatic cancer after first-line gemcitabine-based chemotherapy: a network meta-analysis

SUPPLEMENTARY MATERIALS

Supplementary Table 1: Characteristics of the 8 studies included in the network meta-analysis

Author (year)	Country	Study	BliNRing	Phase	Article	First line		Treatment	Patients	Age	PS			Men (%)	OS [95% CI]	PFS [95% CI]
						GEM (%)	cGEM (%)				0 (%)	1 (%)	2 (%)			
Gillam *et al.* [21] (2016)	Many country	Randomised	open-label	3	Full text	44<br>46	56<br>54	IRI-FP-FA<br>FP-FA	151<br>149	63 (57–70)<br>62 (55–69)	15<br>14	75<br>77	10<br>8	69 (59%)<br>67 (56%)	6.1 [4.8–8.9]<br>4.2 [3.3–5.3]	3.1 [2.7–4.2]<br>1.5 [1.4–1.8]
Gillam *et al.* [21] (2016)	Many country	Randomised	open-label	3	Full text	45<br>44	55<br>56	IRI<br>FP-FA	117<br>149	65 (58–70)<br>63 (55–69)	15<br>15	76<br>77	9<br>8	87 (58%)<br>81 (54%)	4.9 [4.2–5.6]<br>4.2 [3.6–4.9]	2.7 [2.1–2.9]<br>1.6 [1.4–1.8]
Oettle *et al.* [22] (2014)	Germany	Randomised	open-label	2	Full text	100<br>100	0<br>0	FP-FA<br>OXA-FA-FP	87<br>76	61 (43–78)<br>62 (37–83)	47.6<br>53.9	52.4<br>46.1	0<br>0	48 (57.1%)<br>40 (52.6%)	3.3 [2.7–4.0]<br>5.9 [4.1–7.4]	2.0 [1.6–2.3]<br>2.9 [2.4–3.2]
Gill *et al.* [23] (2016)	Canada	Randomised	open-label	3	Full text	77.8<br>74.1	22.2<br>25.9	FP-FA<br>OXA-FP-FA	54<br>54	67 (48–78)<br>65 (38–82)	18.9<br>13	75.5<br>75.9	5.7<br>11.1	30 (55.6%)<br>31 (57.4%)	9.9 [6.7–16.9]<br>6.1 [3.2–7.1]	2.9 [1.7–5.1]<br>3.1 [1.9–7.2]
Ge *et al.* [24] (2014)	People's Republic of China	Randomised	open-label	2	Full text	NR	NR	FP<br>FP-FA	47<br>45	NR<br>NR	53.2<br>48.9	46.8<br>51.1	0<br>0	NR<br>NR	5.5<br>6.3	1.9<br>3.0
Ueno *et al.* [25] (2016)	Japan	Randomised	open-label	2	Full text	NR	NR	FP<br>FP-FA	71<br>69	63.3<br>64.2	67.6<br>65.2	32.4<br>34.8	0<br>0	38 (53.5%)<br>41 (59.4%)	6.1<br>6.3	2.7<br>3.8
Ioka *et al.* [26] (2017)	Japan	Randomised	NR	2	Abstract	NR	NR	FP<br>FP-IRI	127	NR<br>NR	NR<br>NR	NR<br>NR	NR<br>NR	NR<br>NR	5.8<br>6.8	1.9<br>3.5
Mizuno *et al.* [27] (2013)	Japan	Randomised	NR	2	Abstract	NR	NR	FP<br>FP-IRI	67<br>60	NR<br>NR	NR<br>NR	NR<br>NR	NR<br>NR	NR<br>NR	5.9<br>6.9	1.9<br>3.6
Ohkawa *et al.* [28] (2015)	Japan	Randomised	open-label	2	Full text	NR	NR	FP<br>OXA-FP	134<br>130	63.5<br>65	69.4<br>70.8	30.6<br>29.2	0<br>0	80 (61.5%)<br>82 (61.2%)	6.9<br>7.4	2.8<br>3.0

Abbreviations: GEM = gemcitabine, cGEM = gemcitabine combination, IRI = Irinotecan, FP = fluoropyrimidine, FA = Folinic Acid, OXA = Oxaliplatin, OS = Overall Survival, PFS = Progression Free Survival, NR = not reported, PS = performance status.
